# Supplementary material for: Causal Relationship between Aging and Anorexia Nervosa: A White-Matter-Microstructure-Mediated Mendelian Randomization Analysis
Source: Biomedicines. 2024 Aug 16;12(8):1874. doi: 10.3390/biomedicines12081874 (PMC11351342; doi:10.3390/biomedicines12081874)
Supplement: Supplementary file 1 [file biomedicines-12-01874-s001.zip › Supplementary Figure/Figure S4/Singe SNP plot of MR analysis for TL to ubm-b-1777.pdf]

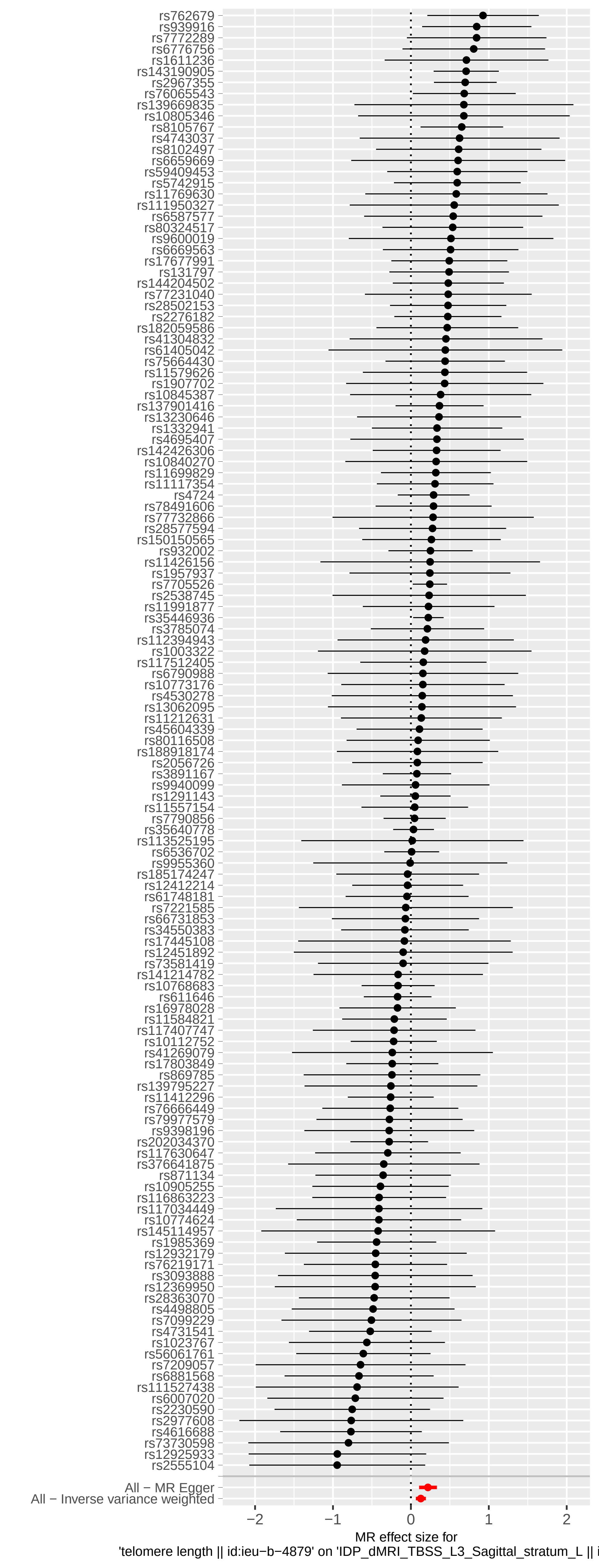
rs762679
rs939916
rs7772289
rs6776756
rs1611236
rs143190905
rs2967355
rs76065543
rs139669835
rs10805346
rs8105767
rs4743037
rs8102497
rs6659669
rs59409453
rs5742915
rs11769630
rs111950327
rs6587577
rs80324517
rs9600019
rs6669563
rs17677991
rs131797
rs144204502
rs77231040
rs28502153
rs2276182
rs182059586
rs41304832
rs61405042
rs75664430
rs11579626
rs1907702
rs10845387
rs137901416
rs13230646
rs1332941
rs4695407
rs142426306
rs10840270
rs11699829
rs11117354
rs4724
rs78491606
rs77732866
rs28577594
rs150150565
rs932002
rs11426156
rs1957937
rs7705526
rs2538745
rs11991877
rs35446936
rs3785074
rs112394943
rs1003322
rs117512405
rs6790988
rs10773176
rs4530278
rs13062095
rs11212631
rs45604339
rs80116508
rs188918174
rs2056726
rs3891167
rs9940099
rs1291143
rs11557154
rs7790856
rs35640778
rs113525195
rs6536702
rs9955360
rs185174247
rs12412214
rs61748181
rs7221585
rs66731853
rs34550383
rs17445108
rs12451892
rs73581419
rs141214782
rs10768683
rs611646
rs16978028
rs11584821
rs117407747
rs10112752
rs41269079
rs17803849
rs869785
rs139795227
rs11412296
rs76666449
rs79977579
rs9398196
rs202034370
rs117630647
rs376641875
rs871134
rs10905255
rs116863223
rs117034449
rs10774624
rs145114957
rs1985369
rs12932179
rs76219171
rs3093888
rs12369950
rs28363070
rs4498805
rs7099229
rs4731541
rs1023767
rs56061761
rs7209057
rs6881568
rs111527438
rs6007020
rs2230590
rs2977608
rs4616688
rs73730598
rs12925933
rs2555104
All − MR Egger
All − Inverse variance weighted
−2
−1
0
1
2
MR effect size for
'telomere length || id:ieu−b−4879' on 'IDP_dMRI_TBSS_L3_Sagittal_stratum_L || i
